# Composite pheochromocytoma–ganglioneuroma: a case with two distinct components radiographically

**DOI:** 10.1259/bjrcr.20220079

**Published:** 2022-09-12

**Authors:** Hiromi Edo, Eiko Hyoue, Kohei Hamamoto, Masaki Tsuda, Fumio Morimura, Kousuke Okano, Michiko Okazaki, Kazuki Kawamura, Keiichi Ito, Kimiya Sato, Naoki Edo, Hiroshi Shinmoto

**Affiliations:** 1 Department of Radiology, National Defense Medical College, Saitama, Japan; 2 Department of Urology, National Defense Medical College, Saitama, Japan; 3 Department of Basic Pathology, National Defense Medical College, Saitama, Japan; 4 Division of Behavioral Science, National Defense Medical College Research Institute, Saitama, Japan; 5 Department of Clinical Research Medicine, Teikyo University School of Medicine, Tokyo, Japan

## Abstract

Composite pheochromocytoma is an extremely rare tumor that comprises a pheochromocytoma and an embryologically related neurogenic tumor, such as ganglioneuroma, ganglioneuroblastoma, neuroblastoma, or peripheral nerve sheath tumor.

A 46-year-old male with hypertension, elevated plasma catecholamine levels, and suspected pheochromocytoma presented to the National Defense Medical College Hospital. CT and MRI showed two adjacent masses in the left adrenal gland; one was a 6 cm cephalic lesion and the other was a 1.5 cm caudal lesion. Only the 1.5 cm caudal mass showed uptake on ^123^I-metaiodobenzylguanisine single photon emission CT/CT. Pheochromocytoma was suspected and a left adrenalectomy was performed. Pathology confirmed that the 6 cm mass was a ganglioneuroma and the 1.5 cm mass a pheochromocytoma, with cellular intermingling at their border. The two masses were diagnosed as a composite pheochromocytoma–ganglioneuroma. This is the first report in which the two components of a composite pheochromocytoma can be clearly distinguished in the pre-operative images. If a patient with clinically suspected pheochromocytoma has different components from a typical pheochromocytoma, composite pheochromocytoma should be considered.

## Clinical presentation

A 46-year-old male with no relevant medical history visited a primary care clinic for a headache that had persisted for a month. Clinical and laboratory investigations revealed hypertension (161/114 mmHg) and elevated plasma catecholamine levels. With a provisional diagnosis of pheochromocytoma/paraganglioma, he visited our hospital. At our hospital, laboratory investigations revealed elevated plasma noradrenaline (1,010 pg/ml) and dopamine (24 pg/ml) levels; although 24 h urinary metanephrine and normetanephrine levels were within the normal range (0.08 mg/day and 0.36 mg/day, respectively), an insufficient suppression of plasma catecholamines was observed in a clonidine suppression test. These findings suggested the presence of a pheochromocytoma/paraganglioma, and imaging examinations were performed.

## Investigations

**Figure 1.**
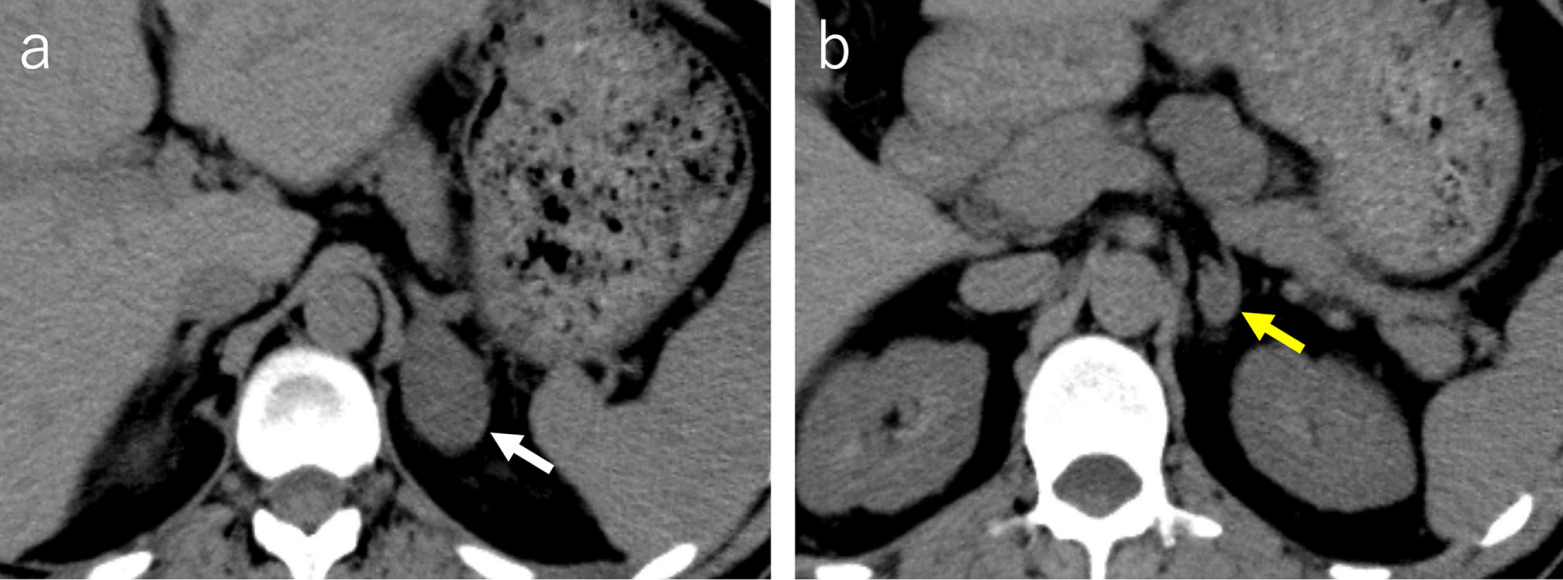
Plain computed tomography (CT) images. (a-, b) CT demonstrates the 6 cm cephalic lesion (white arrow) and the 1.5 cm caudal lesion (yellow arrow) in the left adrenal gland.

**Figure 2.**
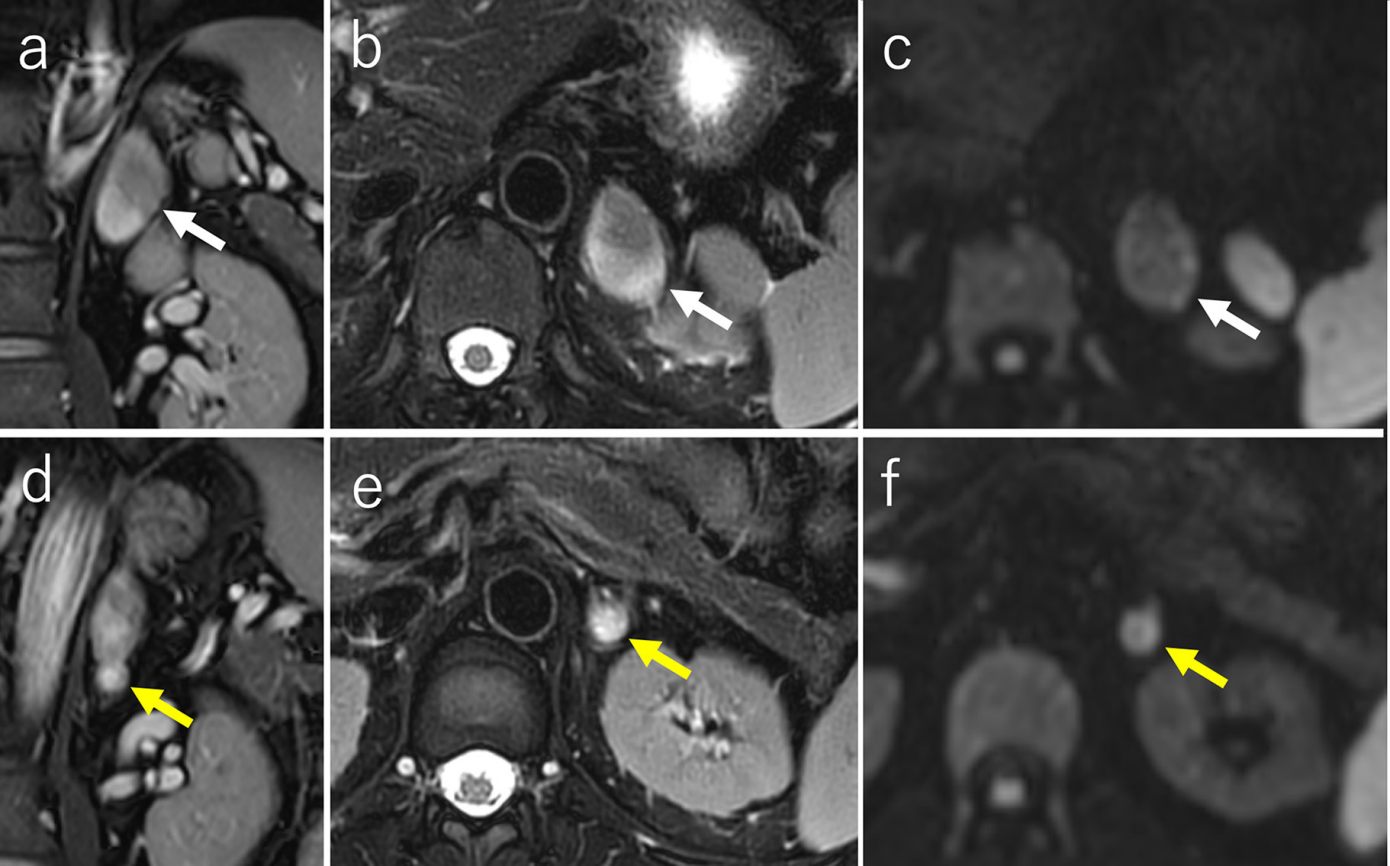
MRI. (**a–c**) MRI of the cephalic mass in the left adrenal gland (white arrows). Coronal (**a**) and axial (**b**) fat-suppressed, T2 weighted images show the cephalic lesion with a mixture of high and low signal intensities and curvilinear bands of low intensity. (**c**) Diffusion-weighted image shows slightly high signal intensity. (**d–f**) MRI of the caudal nodule of the left adrenal gland (yellow arrows). Coronal (**d**) and axial (**e**) fat-suppressed, T2 weighted images show the caudal nodule with heterogeneous, high signal intensity. (**f**) Diffusion-weighted image shows high signal intensity.

**Figure 3.**
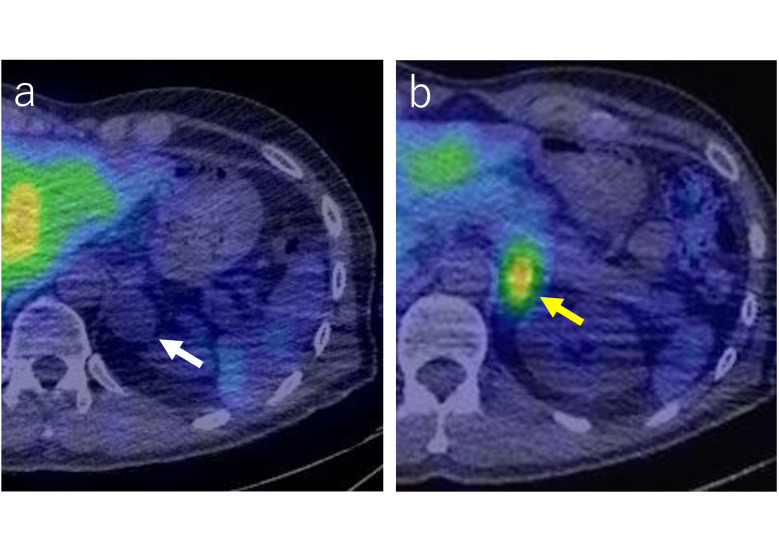
^123^I-metaiodobenzylguanisine (MIBG) single photon emission computed tomography/computed tomography (a) There is no obvious uptake in the cephalic mass in the left adrenal gland (white arrow). (b) MIBG uptake is found in the caudal nodule of the left adrenal gland (yellow arrow).

Plain CT demonstrated two masses in the left adrenal gland: a 6 cm cephalic lesion and a 1.5 cm caudal lesion (Figure 1a and b). On T2 weighted MRI, the cephalic mass demonstrated heterogeneity with a mixture of high and low signal intensities, and the caudal mass demonstrated heterogeneity with high signal intensity (Figure 2a, b, d and e). Diffusion-weighted MRI showed that the cephalic lesion had a slightly high signal intensity, and the caudal lesion had a high signal intensity (Figure 2c and f). Of the two lesions in the left adrenal gland, only the smaller, caudal lesion showed uptake on ^123^I-metaiodobenzylguanisine (MIBG) single photon emission computed tomography (SPECT)/CT (Figure 3a and b).

## Treatment

**Figure 4.**
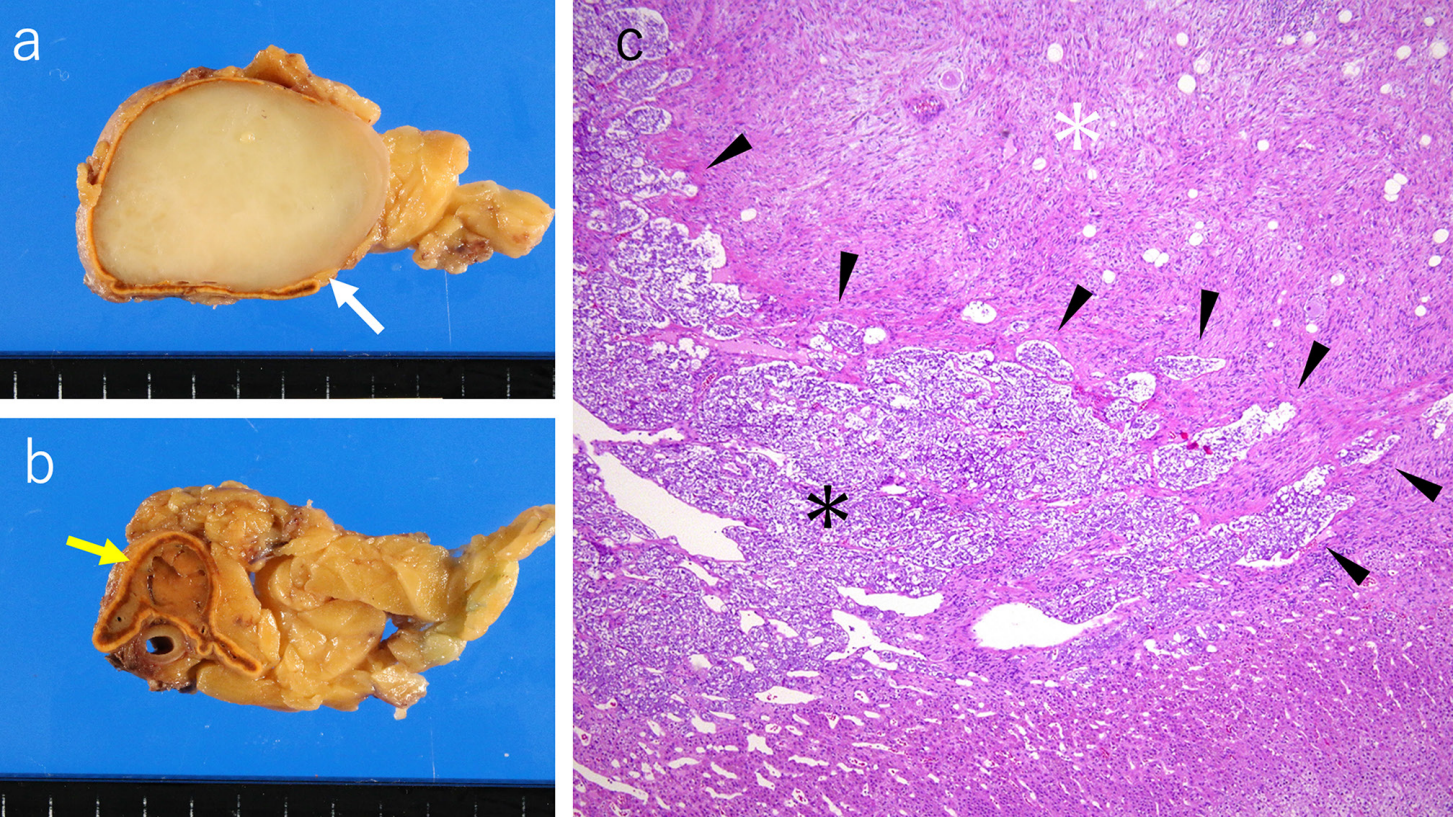
Macroscopic and microscopic images. (**a**) In the gross image, the cephalic mass of the left adrenal gland is seen as a translucent white solid tumor. This is a component of the ganglioneuroma (white arrow). (**b**) In the gross image, the caudal nodule of the left adrenal gland is seen as a reddish-tan solid tumor (a yellow arrow). This is a component of the pheochromocytoma. (**c**) Microscopic examination shows the pheochromocytoma (black asterisk) infiltrating the ganglioneuroma (white asterisk) in bundles and patches at the boundary between the two tumor components, indicating a composite feature (black arrowheads).

Based on the laboratory investigations and pre-operative imaging, pheochromocytoma of the left adrenal gland was suspected, and a left adrenalectomy was performed. The 6 cm cephalic mass was confirmed as a ganglioneuroma, and the 1.5 cm caudal mass was confirmed as a pheochromocytoma based on histopathological examinations Figure 4a and b). At the boundary between the two tumor components, the pheochromocytoma infiltrated the ganglioneuroma in bundles and patches, indicating composite features (Figure 4c. Based on these observations, the tumor was diagnosed as a composite pheochromocytoma–ganglioneuroma.

Retrospectively, MRI and ^123^I-MIBG SPECT/CT demonstrated two lesions in the left adrenal gland, which had different components. Especially in the ^123^I-MIBG SPECT/CT, one lesion showed uptake and the other showed no obvious uptake. There was a history of clinically suspicious findings of pheochromocytoma/paraganglioma, and the caudal mass was consistent with the features of pheochromocytoma based on the MRI and ^123^I-MIBG SPECT/CT. In contrast, the cephalic mass showed heterogeneity with a mixture of high and low signal intensities on T2 weighted imaging, slightly high signal intensity on diffusion-weighted imaging, and no apparent uptake on MIBG scintigraphy, indicating a lesion with different characteristics to those of pheochromocytoma.

### Outcome and follow-up

3 years after the left adrenalectomy, there was no obvious recurrence or metastasis.

## Discussion

To the best of our knowledge, this is the first case report of a composite pheochromocytoma–ganglioneuroma in which each component could be clearly distinguished on pre-operative imaging. Notably, the ganglioneuroma component was larger than the pheochromocytoma component.

When two different neoplasms coexist adjacent to each other in a single organ, the combination is either a “composite tumor” or a “collision tumor.”^1^ Composite tumors are defined as tumors originating from the same stem cells, while collision tumors are defined as tumors originating from different sources.^1^ The most common type of composite tumor in the adrenal gland is pheochromocytoma–ganglioneuroma; both the chromaffin cells and the sympathetic ganglion cells originate from primitive neuroectodermal cells from the neural crest.^2,3^ Usually, composite features and cellular intermingling are first noted microscopically.^2^ Initially, the imaging studies reported here suggested that a collision tumor was more likely than a composite tumor owing to the presence of two adjacent, distinct nodules. However, pathological examination showed cellular infiltration at the boundary of the two tumor components, leading to the diagnosis of a composite tumor.

In our case, the ganglioneuroma was the larger component. Lam et al and Shawa et al reported four and nine cases of composite pheochromocytoma–ganglioneuroma, respectively, all of which had pheochromocytoma as the dominant component.^4,5^ There is no previous report of composite pheochromocytoma–ganglioneuroma in which the ganglioneuroma component was predominant, as seen in the present case.

MIBG uptake is found in 81–85% of pheochromocytomas and is known to be highly specific for diagnosis.^6,7^ In contrast, MIBG uptake is found in 25–57% of ganglioneuromas.^8,9^ This dissimilarity in tumor affinity to MIBG is demonstrated in the differential accumulation between each component. The most common accompanying neurogenic tumor among composite pheochromocytoma is ganglioneuroma (61–65%),^3^ as in this case, but ganglioneuroblastoma and neuroblastoma are also found. The MIBG-uptake rates for ganglioneuroblastoma and neuroblastoma are 61.1 and 89.6%, respectively.^8^ In our case, ^123^I-MIBG SPECT/CT was useful in distinguishing the components of pheochromocytoma and ganglioneuroma. In particular, it was difficult to precisely assess the area of accumulation in the mass with conventional MIBG scintigraphy, whereas ^123^I-MIBG SPECT/CT was useful for such assessment.

Retrospectively, each component was consistent with typical features on MRI. One of the characteristic MRI findings of ganglioneuroma is a whorled appearance, which is caused by curvilinear or nodular bands of low intensity found on T2- and/or T1 weighted imaging.^10,11^ The ganglioneuroma component of the present case had a whorled appearance on T2 weighted imaging. The pheochromocytoma component showed heterogeneous, high signal intensity, suggesting microcystic change, on T2 weighted imaging. On diffusion-weighted imaging, signal intensity of the pheochromocytoma component was higher than that of the ganglioneuroma component. The MRI findings of the two components showed different features. However, it is rarely possible to make a pre-operative diagnosis of composite pheochromocytoma–ganglioneuroma based on MRI findings alone.

In conclusion, we report a composite pheochromocytoma–ganglioneuroma in which the two components could be distinguished on pre-operative MRI and ^123^I-MIBG SPECT/CT. Although it is impossible to distinguish between composite and collision tumors on pre-operative imaging, the possibility of a composite or collision tumor should be considered when two different tumor components are found in a single organ.

## Informed consent statement

Written informed consent was obtained from the patient for publication of this case report, including accompanying images.

## Learning points

1. When two adjacent tumor lesions with different components are found in a single organ, a composite or collision tumor should be considered.
2. In patients in whom clinical and hormonal investigations point to pheochromocytoma and imaging shows two adjacent lesions in the adrenal gland, the radiologists should consider a composite pheochromocytoma or a collision tumor combining pheochromocytoma with any other neoplasm as a differential diagnosis.
